# Supplementary material for: The Effect of Hyaluronic Acid and Chondroitin Sulphate-Based Medical Device Combined with Acid Suppression in the Treatment of Atypical Symptoms in Gastroesophageal Reflux Disease
Source: J Clin Med. 2022 Mar 29;11(7):1890. doi: 10.3390/jcm11071890 (PMC9000081; doi:10.3390/jcm11071890)
Supplement: Supplementary file 1 [file jcm-11-01890-s001.zip › jcm-1654580-supplementary.pdf]

Table S1. Summary of results regarding the changes in individual RSI item scores between baseline and any time point during the 6-week treatment period (no statistically different items).

Item	Treatment	Visit	n	Mean	SD	Median	Min	Max
Difficulty swallowing food, liquids or pills	Gerdoff®+omeprazole	Visit 1 Baseline/Screening (T0)	35	1.9	1.66	2	0	5
		Visit 2 (T1): 1 week ± 1 day after baseline	32	0.9	1.27	0	0	4
		Visit 3 (T3): 3 weeks ± 2 days after baseline	28	0.8	1.13	0	0	5
		Visit 4 (T6): 6 weeks ± 2 days after baseline	31	0.4	0.71	0	0	2
	Omeprazole	Visit 1 Baseline/Screening (T0)	36	1.9	1.84	1.5	0	5
		Visit 2 (T1): 1 week ± 1 day after baseline	35	1.1	1.37	1	0	5
		Visit 3 (T3): 3 weeks ± 2 days after baseline	35	1.1	1.48	0	0	5
		Visit 4 (T6): 6 weeks ± 2 days after baseline	36	0.6	0.94	0	0	3
Coughing after eating or lying down	Gerdoff®+omeprazole	Visit 1 Baseline/Screening (T0)	35	2.5	1.76	2	0	5
		Visit 2 (T1): 1 week ± 1 day after baseline	32	1.5	1.70	1	0	5
		Visit 3 (T3): 3 weeks ± 2 days after baseline	28	1.2	1.16	1	0	4
		Visit 4 (T6): 6 weeks ± 2 days after baseline	31	0.7	1.01	0	0	3
	Omeprazole	Visit 1 Baseline/Screening (T0)	36	3.3	1.44	3.5	0	5
		Visit 2 (T1): 1 week ± 1 day after baseline	35	2.5	1.34	3	0	5
		Visit 3 (T3): 3 weeks ± 2 days after baseline	35	1.7	1.36	2	0	5
		Visit 4 (T6): 6 weeks ± 2 days after baseline	36	1.7	1.47	1.5	0	5
Breathing difficulties or choking episodes	Gerdoff®+omeprazole	Visit 1 Baseline/Screening (T0)	35	1.5	1.60	1	0	5
		Visit 2 (T1): 1 week ± 1 day after baseline	32	0.8	1.35	0	0	4
		Visit 3 (T3): 3 weeks ± 2 days after baseline	28	0.6	1.07	0	0	4
		Visit 4 (T6): 6 weeks ± 2 days after baseline	31	0.5	1.15	0	0	5

	Omeprazole	Visit 1 Baseline/Screening (T0)	36	1.9	1.69	2	0	5
		Visit 2 (T1): 1 week ± 1 day after baseline	35	0.7	1.15	0	0	4
		Visit 3 (T3): 3 weeks ± 2 days after baseline	35	0.8	1.15	0	0	4
		Visit 4 (T6): 6 weeks ± 2 days after baseline	36	0.6	0.97	0	0	3
Troublesome or annoying cough	Gerdoff®+omeprazole	Visit 1 Baseline/Screening (T0)	35	2.5	1.65	3	0	5
		Visit 2 (T1): 1 week ± 1 day after baseline	32	1.9	1.58	2	0	5
		Visit 3 (T3): 3 weeks ± 2 days after baseline	28	1.6	1.59	1	0	5
		Visit 4 (T6): 6 weeks ± 2 days after baseline	31	0.8	1.17	0	0	5
	Omeprazole	Visit 1 Baseline/Screening (T0)	36	3.7	1.19	4	0	5
		Visit 2 (T1): 1 week ± 1 day after baseline	35	2.4	1.61	2	0	5
		Visit 3 (T3): 3 weeks ± 2 days after baseline	35	1.9	1.46	2	0	5
		Visit 4 (T6): 6 weeks ± 2 days after baseline	36	1.5	1.54	1	0	5
Sensation of something sticking in the throat/lump in the throat	Gerdoff®+omeprazole	Visit 1 Baseline/Screening (T0)	35	3.0	1.52	3.0	0	5
		Visit 2 (T1): 1 week ± 1 day after baseline	32	1.5	1.65	1.0	0	5
		Visit 3 (T3): 3 weeks ± 2 days after baseline	28	1.6	1.55	1.5	0	5
		Visit 4 (T6): 6 weeks ± 2 days after baseline	31	0.8	1.34	0.0	0	5
	Omeprazole	Visit 1 Baseline/Screening (T0)	36	2.6	1.75	3.0	0	5
		Visit 2 (T1): 1 week ± 1 day after baseline	35	2.3	1.51	2.0	0	5
		Visit 3 (T3): 3 weeks ± 2 days after baseline	35	1.7	1.54	2.0	0	5
		Visit 4 (T6): 6 weeks ± 2 days after baseline	36	1.2	1.45	1.0	0	5
Heartburn, chest pain, indigestion or stomach acid in the mouth	Gerdoff®+omeprazole	Visit 1 Baseline/Screening (T0)	35	3.4	1.56	4	0	5
		Visit 2 (T1): 1 week ± 1 day after baseline	32	2.0	1.40	2	0	4
		Visit 3 (T3): 3 weeks ± 2 days after baseline	28	1.5	1.45	1	0	5
		Visit 4 (T6): 6 weeks ± 2 days after baseline	31	0.8	1.08	0	0	3

Omeprazole	Visit 1 Baseline/Screening (T0)	36	3.5	1.83	4	0	5
	Visit 2 (T1): 1 week ± 1 day after baseline	35	2.4	1.75	3	0	5
	Visit 3 (T3): 3 weeks ± 2 days after baseline	35	1.8	1.59	1	0	5
	Visit 4 (T6): 6 weeks ± 2 days after baseline	36	1.6	1.59	1	0	5

n = number of observations.

Table S2. Results of distribution of patient's satisfaction with treatment.

Treatment	Visit	Opinion	n	%
Gerdoff®+ omeprazole	Visit 2 (T1): after 1 weeks from baseline ± 1 day	Low	2	6.3
		Discrete	4	12.5
		Good	13	40.6
		Excellent	13	40.6
	Visit 3 (T3): after 3 weeks from baseline ± 2 days	Low	1	3.6
		Discrete	2	7.1
		Good	11	39.3
		Excellent	14	50.0
	Visit 4 (T6): after 6 weeks from baseline ± 2 days	Missing	3	-
		Low	3	10.0
		Discrete	1	3.3
		Good	11	36.7
		Excellent	15	50.0
Omeprazole	Visit 2 (T1): after 1 weeks from baseline ± 1 day	Low	3	8.6
		Discrete	10	28.6
		Good	11	31.4
		Excellent	11	31.4
	Visit 3 (T3): after 3 weeks from baseline ± 2 days	Low	1	2.9
		Discrete	5	14.3
		Good	15	42.9
		Excellent	14	40.0
	Visit 4 (T6): after 6 weeks from baseline ± 2 days	Low	1	2.8
		Discrete	7	19.4
		Good	11	30.6
		Excellent	17	47.2

n = number of observations.
